# Improving the Diagnosis of Rib Fractures in the Emergency Department: A Quality Improvement Project in a District General Hospital in the United Kingdom

**DOI:** 10.7759/cureus.56873

**Published:** 2024-03-25

**Authors:** Salman Naeem, Shadman Aziz, Fernando Carballido, Jonathan Leung

**Affiliations:** 1 Emergency Medicine, East Kent Hospitals University NHS Foundation Trust, Ashford, GBR; 2 Emergency Medicine, Barts Health NHS Trust, London, GBR; 3 Prehospital Emergency Medicine, East Anglian Air Ambulance, Norwich, GBR; 4 Prehospital Emergency Medicine, Air Ambulance Kent, Surrey and Sussex, Redhill, GBR

**Keywords:** rib fractures, silver trauma, emergency department, quality improvement, trauma

## Abstract

Background

Falls in older people are a common presentation in emergency departments (ED) in the United Kingdom. They can lead to multiple injuries, including chest wall injuries (CWIs). Untreated CWI carries significant morbidity and mortality. However, its diagnosis remains challenging during the initial ED encounter. This led to a quality improvement project (QIP) to improve the diagnosis of CWI in patients presenting to William Harvey Hospital, a district general, trauma-unit ED in Willesborough, England.

Methods

The QIP was run from February 2020 to April 2021 for 14 months. A series of plan-do-study-act (PDSA) cycles were completed to increase the proportion of CWIs diagnosed during the initial ED encounter to 90%. The primary interventions involved designing a new thoracic trauma proforma and the introduction of the modified pain, inspiratory effort, and cough (PIC) score to evaluate and triage patients with CWI. Other interventions included the delivery of an education programme on CWI. The secondary aims were to increase modified PIC score use and to reduce the time between ED presentation and computerised tomography (CT) scanning.

Results

A total of 147 patients were included in three PDSA cycles. The diagnosis of CWI during the initial ED encounter increased from 61% at baseline to 91%. The median time from ED attendance to the first CT reduced from 477 minutes to 169 minutes. Lastly, following the introduction of the thoracic trauma proforma, the modified PIC score was used in 26% of cases of CWI by the end of the QIP period.

Conclusion

Our QIP led to improvement in the early diagnosis of CWIs in ED, with significant improvements in door to CT time and the creation of a thoracic injury pathway in the trust leading to multi-specialty improvement of care of such patients.

## Introduction

Falls in older people are a common presentation in emergency departments (ED) in the United Kingdom [1]. According to UK government statistics, a third of patients above 65 years and about half of patients above 80 years will fall once a year [2]. Falls from standing often lead to multiple injuries in older people, most commonly to the head and thoracic cavity [3].

Thoracic trauma accounts for almost 10-15% of presentations to EDs worldwide [4]. It has a significantly high mortality that increases with age, reaching up to 22% in patients above 65 years [5]. Mortality and morbidity are due to the increased risk of developing pneumonia and acute respiratory distress syndrome (ARDS) due to reduced ventilation, poor cough effectiveness, and reduced secretion clearance due to pain [6]. Early diagnosis and management can lead to reduced morbidity and mortality [7].

This quality improvement project (QIP) was done at William Harvey Hospital (WHH), an acute district general hospital in Willesborough, England. It is one of the three hospitals and the only level 2 trauma unit in the trust. It has a busy ED with over 130,000 adult and paediatric presentations per year. Prior to the development of this QIP, the project team became aware of multiple patients with delayed or missed diagnosis of thoracic trauma, who subsequently deteriorated onwards.

The project team investigated various factors leading to the missed diagnosis of chest wall injury (CWI) at the hospital. They found that anecdotally, local clinician knowledge about the risk of CWI in elderly fallers, and the risk of mortality with it, was poor. It was also highlighted that the existing chest trauma pathway for patients at the hospital utilised outdated evidence and required updating.

The original trust pathway for the diagnosis of thoracic injury in non-major trauma patients recommended plain chest X-ray as the initial diagnostic imaging modality. However, updated literature suggested that chest X-rays were unreliable for the diagnosis of rib fractures, missing up to 45% of fractures [8]. In 2018, the London Major Trauma Service published their guidance on the management of major trauma in elderly patients which highlighted the issue of CWI in the elderly and the associated morbidity and mortality [1]. The guidance recommended the early recognition of CWI, low threshold for contrast CT, multidisciplinary care for elderly trauma patients, and development and implementation of local CWI bundles.

However, the hospital has only two computerised tomography (CT) scanners that cater to the whole hospital. Only one CT scanner is available out-of-hours, and the other is also used for outpatient scanning in hours. Consequently, due to the high proportion of elderly patients presenting to the ED, it is difficult to perform a timely CT scan in all patients presenting with a fall or suspected CWI. Therefore, it was recognised that there was a need to risk-stratify, identify, and triage those patients who require a CT scan.

There is literature on risk factors associated with mortality following rib fractures, and various tools have been developed for prognostication after the diagnosis of CWI [4,9]. The NEXUS Chest Decision Instrument is an excellent tool to rule out significant CWI and the need for imaging low-risk patients [10]. However, after an extensive literature search, we were unable to find a tool that would stratify the risk of major chest injury or help identify those likely to have rib fractures in the ED setting. In the absence of such a tool, the literature search was expanded to include patient monitoring tools post-CWI.

The pain, inspiratory effort, and cough (PIC) score was originally developed to reduce unplanned intensive care admissions in blunt chest trauma patients [11]. It assesses the adequacy of ventilation and gives an estimation of the risk of developing a chest infection. We postulated that this score could be used to identify and risk-stratify patients with potential for CWI. However, since it had been used as a monitoring tool for patients with rib fractures in the intensive care setting, it was difficult to use spirometry for the assessment of breathing in the ED. Therefore, we modified the PIC score by inverting the scoring of severity so that patients with severe limitations in their ventilatory effort and pain would score the highest and also made the assessment of breathing subjective to treating clinicians' interpretation. We sought to use this score to improve the diagnosis of CWI and to expedite cross-sectional imaging.

As a result, a QIP was developed to improve the diagnosis of CWI at the hospital. The project's SMART aim was to correctly diagnose CWI following initial assessment in ED in 90% of patients with CWI by the end of 12 months of the QIP period.

## Materials and methods

Measurement

All patients with a diagnosis of CWI at any point during their hospital admission during the QIP period were included. Patients with incomplete documents were excluded. A retrospective data collection was done for the initial audit which included all patients admitted with CWI from January 2019 to July 2019 from the William Harvey Hospital database.

Patient notes were reviewed to establish whether the diagnosis of CWI occurred whilst patients were in ED or afterwards (e.g., in the clinical decision unit, on in-patient wards, or following representation to ED if discharged). The initial audit identified 77 patients who suffered CWI. In 61% (n=47) of patients, ED clinicians missed the diagnosis of CWI injuries during their initial consultation. The average time from attendance to the first CT was 477 minutes.

A SMART aim was devised to correctly diagnose CWI following initial assessment in the ED in 90% of patients with CWI by the end of 12 months of the QIP period. The primary outcome measure was the percentage of patients correctly diagnosed with CWI during initial assessment in ED in those admitted to the hospital for chest injuries. The process measure was door to CT time. The use of the modified PIC score (established by measuring the use of a thoracic trauma proforma) was a balance measure.

Ethical considerations

This project did not meet the UK Health Research Authority criteria for research. Ethical approval was therefore not required, and it was registered locally as a QIP. Patients were not involved in the planning or design of this project. The manuscript was prepared following SQUIRE (Standards for QUality Improvement Reporting Excellence) V2.0 guidelines [12].

Design

The QIP team involved an emergency medicine consultant (JL), ED nurse (FC), and emergency medicine trainee (SA) and was led by an emergency medicine higher specialty trainee (SN). JL, FC, and SN designed the project. Data collection and analysis were completed by SN. SN wrote the first draft, whilst the first and subsequent drafts were written and critically revised by SA.

**Figure 1:**
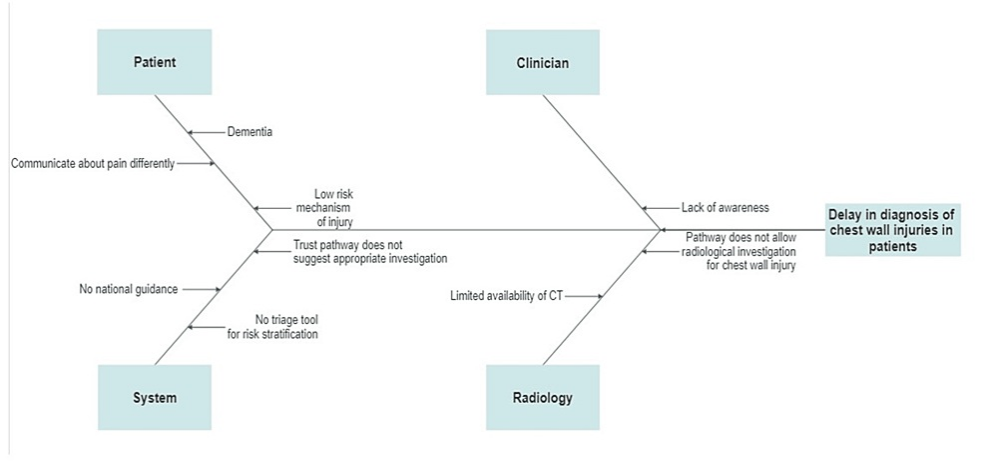
Fishbone analysis diagram Fishbone analysis diagram showing various elements assessed for missed diagnosis of chest wall injury in the emergency department. Image Credit: Salman Naeem

The QIP was done prospectively from February 2020 to April 2021 for 14 months. The data for all the plan-do-study-act (PDSA) cycles, i.e., PDSA 1, 2, and 3, were collected retrospectively from the WHH database. To plan the QIP interventions, a root cause analysis using a patient who had a missed CWI as an example was conducted. The patient's journey through ED to the point of diagnosis of CWI was mapped by establishing a timeline of events. Fishbone analysis was then used to find the causes for missed diagnosis of CWI (Figure 1). It identified a need to streamline and expedite the diagnosis of CWI in patients presenting to ED.

**Figure 2:**
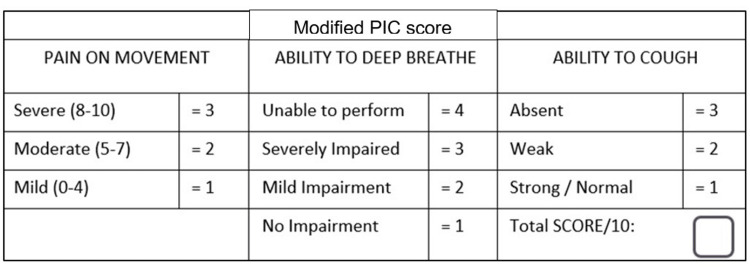
Modified PIC score The modified PIC score is a modified version of the PIC score. It consists of 10 points based on pain on movement, ability to deep breathe, and ability to cough. Pain is categorised into three categories based on pain score on the visual analogue scale. Ability to deep breathe is assessed subjectively based on the assessment of the physician and categorised into four categories. Ability to cough is also assessed subjectively based on the assessment of the physician and categorised into three categorised. The scoring is done in ascending order for increasing severity of the modified PIC score. PIC: pain, inspiratory effort, and cough Image Credit: Salman Naeem

The first planned intervention was the introduction of a triage tool that would risk-stratify patients with a chest injury. The original PIC score [11] was modified (Figure 2) and used to stratify the patients at risk of CWI. We considered its use as it gauged the degree of ventilatory compromise due to pain, which is one of the main causes of morbidity and mortality in CWI patients [11]. Patients with no ventilatory compromise scored a minimum of 3 points, and patients with maximum compromise scored 10 points. We used the discharge score of 5 points used in the original study as the score that would trigger a clinician to think about significant CWI [11].

The second intervention was to introduce criteria to identify patients who required a chest CT with contrast to diagnose the extent of their injury. The aim of this was to rationalise the use of CT and improve access in patients with high-risk features. Given the limitation of availability of CT in our hospital and the high volume of elderly falls presenting to our ED, we created criteria to specify who required chest CT with contrast which included high-risk mechanism of injury (as per the Advanced Trauma Life Support guidelines) [13], penetrating chest injury, and frail/aged >65 with a PIC score of ≥5 +/- blunt chest trauma with clinical signs of injury.

This was included in a thoracic trauma pathway form that was adapted from the chest trauma pathway being used at Warrington and Halton Hospitals NHS Trust (Appendix A). A supporting chest trauma algorithm for adults was created to specify the management of such patients (Appendix B). The proforma and algorithm were approved by hospital lead clinicians for ED, trauma, pain, intensive care, and radiology.

The third intervention was to improve clinicians' knowledge and awareness about high-risk patients for chest trauma, the importance of early diagnosis and pain management, limitations of plain radiography, and complications associated with chest trauma. Previous QIPs have found that a structured educational programme and frequent reminders to busy clinicians in ED can increase compliance and completion of proformas [14]. The educational programme involved introducing short teaching sessions to the morning handover, delivering teaching sessions in the doctor's monthly teachings, putting up posters at various places in the ED, and, lastly, emailing reminders to the doctors in the ED.

Strategy

A series of PDSA cycles were designed. Patient data was retrospectively collected for all PDSA cycles.

PDSA 1

The paper-based thoracic injury pathway proforma was introduced to the ED team in February 2020. The proforma included the modified PIC score for the identification of patients with potential CWI, the criteria for chest CT with contrast, diagrams of the thoracic cage to highlight injuries, rib fracture score for prognostication [15], guidance on the further management of patients including admission criteria, transfer criteria to major trauma centre, and a pain management algorithm. The proforma was designed to be user-friendly and concise, sequentially detailing steps for patient management from diagnosis through to admission/transfer and pain management. This was done to minimise the time required for the completion of the proforma whilst guiding the clinician through every step of management.

The project team attended morning and afternoon handovers and monthly ED teaching sessions, and posters were put in the ED during the first PDSA cycle to make all the ED doctors aware of the project and proforma. Informal feedback was also collected about doctors' knowledge about chest trauma, the awareness of the new proforma, and ease of use.

PDSA cycle 1 ran from March 2020 to May 2020. Although the initial plan was to run a PDSA cycle each month, there was a lag of about two months in extracting data from the hospital database; hence, each PDSA cycle was then run for three months. The QIP was further delayed due to the coronavirus disease 2019 (COVID-19) pandemic which also started in March 2020.

PDSA 2

PDSA cycle 2 ran from August 2020 to October 2020. It was noted that the uptake of the proforma was not very good. A formal feedback survey was sent to ED clinicians which highlighted the following issues. Around 73% of the clinicians were still using clinical gestalt to stratify risk for injury. Around 42% still identified chest X-ray as the investigation of choice in chest trauma. Around 90% indicated the correct use of chest trauma pathway in suspected chest trauma patients. For factors inhibiting chest trauma pathway proforma use, 47% highlighted that it was not readily available, 26% were not aware of its existence, 16% did not know how to use it, and 10% did not think it was clinically relevant. Clinicians also gave suggestions to improve the proforma and its use.

Interventions were then introduced to improve awareness, use, and knowledge about the chest trauma pathway proforma in ED. Firstly, the project team delivered mini-teaching sessions in the morning and afternoon handovers of doctors, presented at monthly teaching sessions, and sent email reminders. However, the COVID-19 pandemic continued to limit the opportunity to teach, as staff were overwhelmed with numerous changes in the flow of patients in the department, and combined handovers and teaching were cancelled due to social distancing. Secondly, a drawer in resus was allocated to the pathway proforma. A picture of its location was then emailed to the ED staff. Clinicians were also informally shown the location of the drawer whilst on the shop floor. The third intervention was to modify the current trust pathway for chest trauma to allow chest CT with contrast to investigate chest wall trauma. This was done through the agreement with the trust lead for radiology and the ED. Lastly, nursing staff also received teaching on the pathway.

PDSA 3

**Figure 3:**
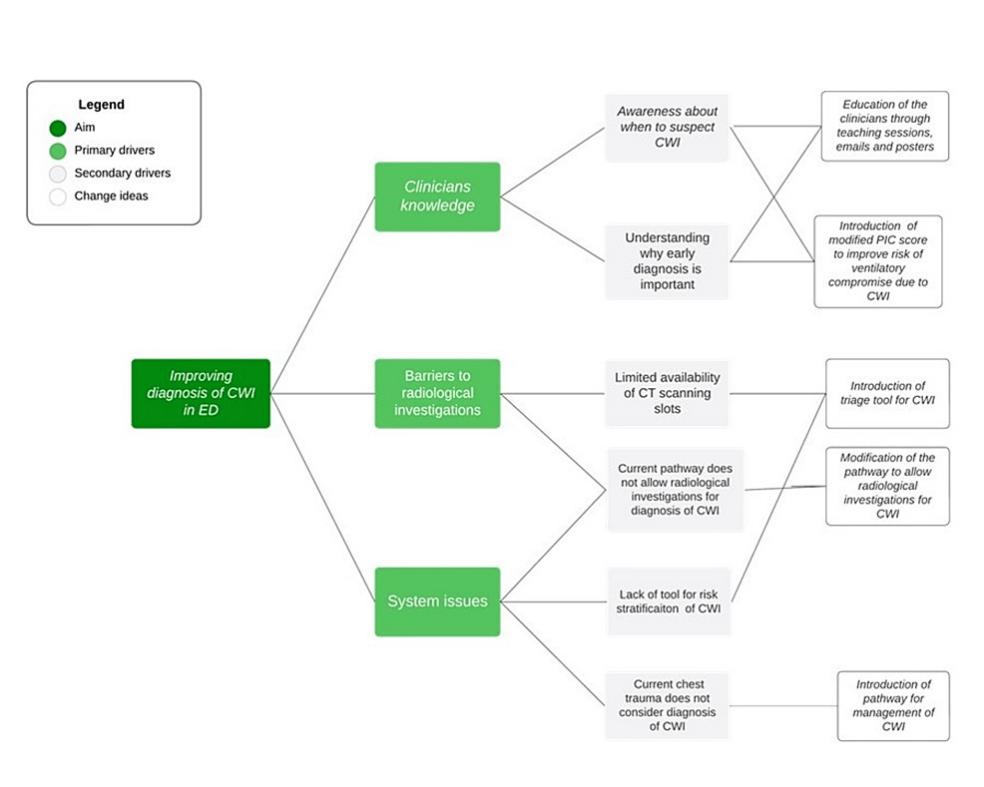
Driver diagram Driver diagram of the quality improvement project showing multiple drivers for delay in the diagnosis of CWI in the emergency department and the interventions introduced in the department. CWI: chest wall injury Image Credit: Salman Naeem

The third PDSA cycle was run from February 2021 to April 2021. There was a delay in the initiation of this cycle due to the second wave of the COVID-19 pandemic in the United Kingdom. Informal teaching sessions, posters, and email reminders were increased to keep staff aware of the chest trauma pathway proforma. Junior doctors who started rotation in the department were introduced to the pathway and the use of modified PIC score during their induction. However, interventions during this PDSA cycle were limited to social distancing and lockdown during the COVID-19 pandemic. This was done intentionally as we did not want to overburden already-stressed staff about changes in the department. Figure 3 summarises the primary and secondary drivers and corresponding interventions.

**Figure 4:**
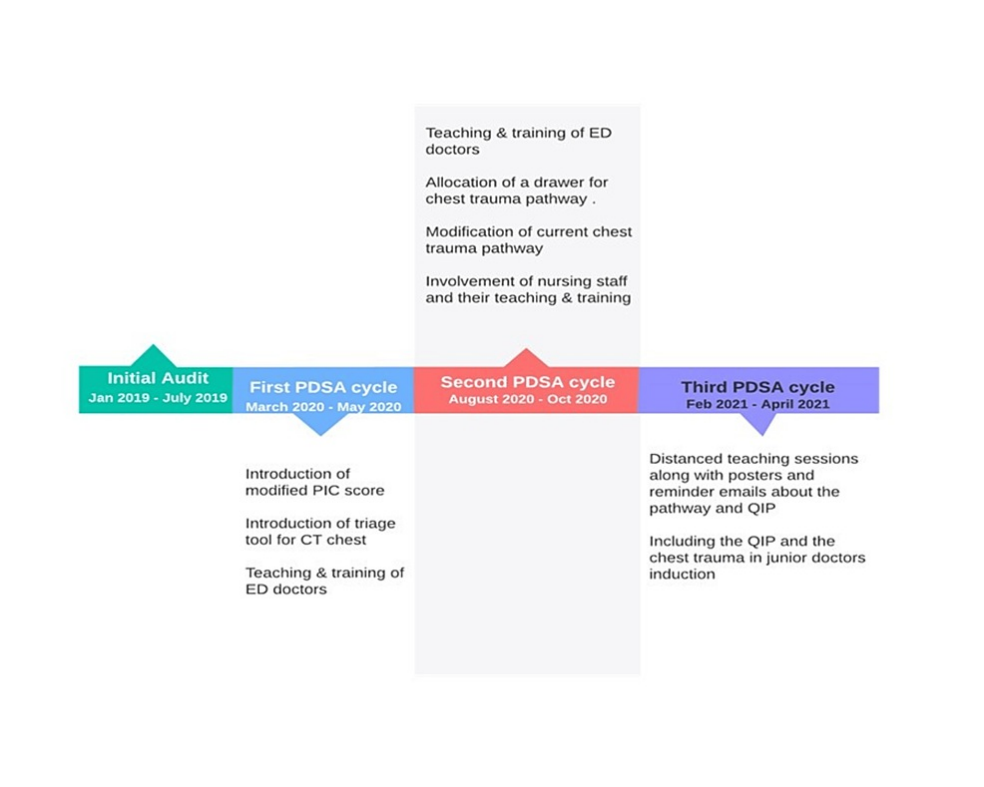
Timeline of the quality improvement project Timeline of the quality improvement project showing interventions done during each PDSA cycle. PDSA: plan-do-study-act Image Credit: Salman Naeem

Figure 4 summarises the timeline of the QIP.

## Results

**Figure 5:**
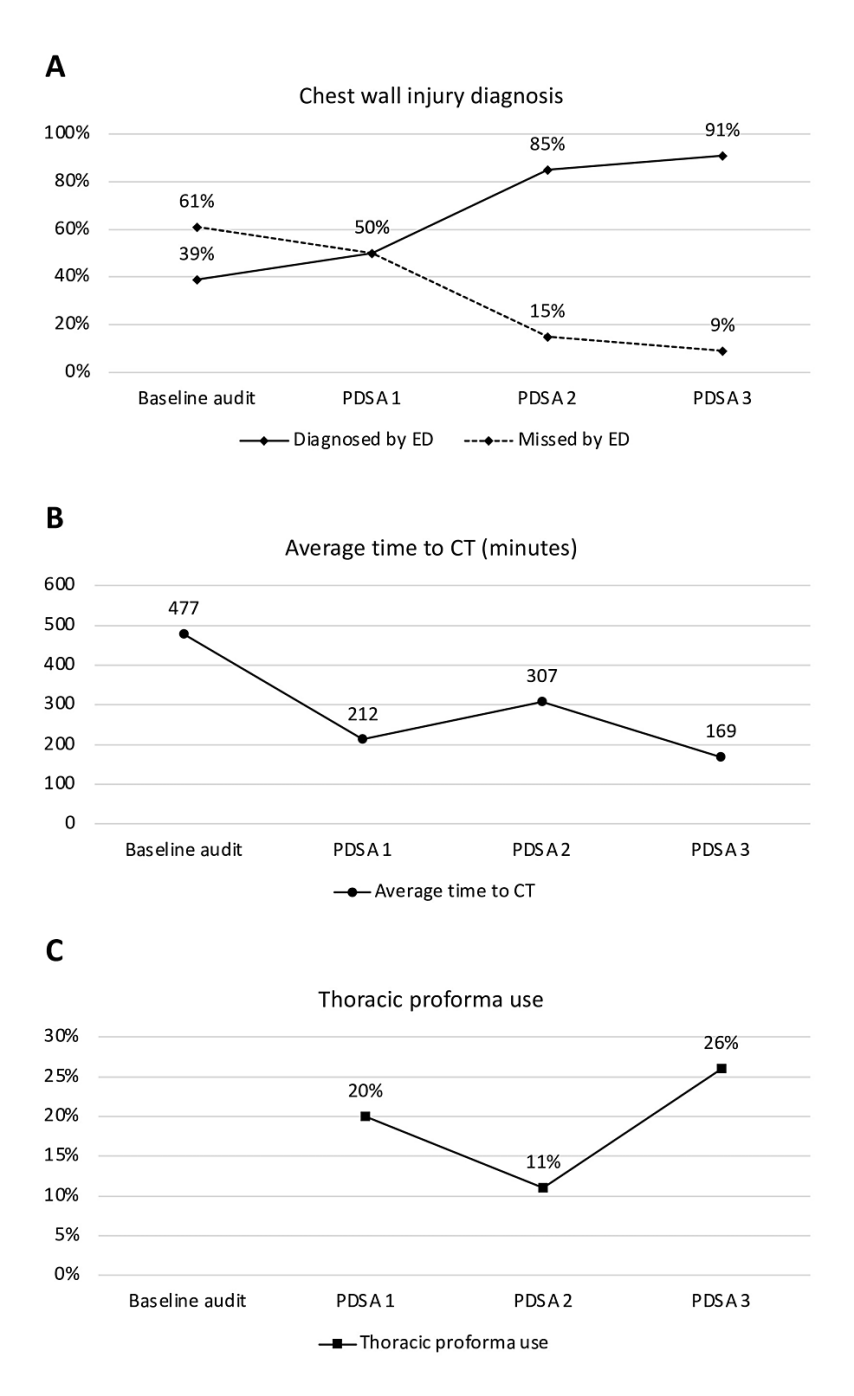
Run charts of the QIP Figure 5A shows the percentage of diagnosed CWI during the initial ED encounter throughout the QIP represented by the solid line and the percentage of missed CWI during the initial ED encounter represented by the dotted line. Figure 5B shows the average time to CT from arrival in the ED in minutes over the duration of the QIP. Figure 5C shows the use of thoracic proforma use over the duration of the QIP. CWI: chest wall injury; ED: emergency department; QIP: quality improvement project Image Credit: Salman Naeem

There were a total of 147 patients who had CWI recorded on the WHH database (baseline: 77, PDSA 1: 10, PDSA 2: 26, PDSA 3: 34). Forty-two patients had a missed diagnosis of CWI in ED (baseline: 30, PDSA 1: 5, PDSA 2: 4, PDSA 3: 3). The proportion of patients with missed diagnosis of CWI in ED decreased steadily throughout the study period (Figure 5A). The SMART aim was achieved with 91% of patients being correctly diagnosed with CWI following the initial assessment in WHH ED.

The average time from admission to the first CT was reduced by approximately two-thirds from 477 minutes during the baseline study to 169 minutes after PDSA cycle 3 (Figure 5B). Lastly, thoracic proforma use also increased (PDSA 1: 2, PDSA 2: 3, PDSA 3: 9); however, the proportion of patients with CWI who had a proforma completed fluctuated throughout the study period (Figure 5C).

## Discussion

These results suggest that a structured education programme and introduction of a thoracic trauma proforma utilising the modified PIC score is effective in reducing the number of missed diagnoses of CWI and in reducing the average time to CT. Improvement in the diagnosis of CWI in the ED is likely secondary to raising the awareness of the issue from these interventions. This was reflected in the steady improvement in the proportion of patients diagnosed in each PDSA cycle during the QIP.

Furthermore, the average time to CT was likely reduced due to the multi-specialty buy-in and engagement from diagnostic radiology. Time to CT was also lower when there was a higher proportion of thoracic proforma use, suggesting these two results were correlated. This could be because the proforma might have acted as a prompt for the clinician to request and transfer the patient to CT.

Lastly, the update of the trust thoracic injury pathway ensured the sustainability of the project and encouraged interprofessional involvement including chest physiotherapists and anaesthesia. However, further study is required to investigate the effect of this on patient outcomes.

Lessons and limitations

A major limitation of this QIP was that it was not possible to identify the true number of patients in ED who had a missed diagnosis of CWI. Patients were identified from the hospital database which led to selection bias [16] as these are patients who have eventually been correctly diagnosed with CWI. However, it was infeasible to identify and audit all patients who presented to ED with traumatic CWI.

Furthermore, in the absence of a validated score for the identification of CWI in the ED, a modified version of the PIC score [11] was used to identify and risk-stratify patients with suspected CWI. The PIC score was not developed to be used as a risk stratification tool, as it is used as a monitoring tool in the intensive care setting. Future steps would be to test the performance of the modified PIC score in picking up CWI to validate its use in this context.

In addition, the overall sample size of this project was quite small. This could be due to the first COVID-19 pandemic lockdown [17], which came into effect in March 2020 in the United Kingdom. Additionally, due to social distancing, all educational activities were stopped at WHH. Hence, regular teaching sessions about the CWI pathway and modified PIC score were cancelled which likely impacted the change process. Despite achieving the SMART aim, the COVID-19 pandemic significantly delayed the QIP, which meant that the original 12-month target deadline was unfortunately not met.

This QIP was conducted in a single ED in the United Kingdom; hence, the QIP cannot be generalised to other EDs. The modified PIC score has not been externally validated. Lastly, during the QIP period, the trust changed its electronic patient record from Allscripts to Sunrise in November 2020. This meant that it was not possible to create a digital proforma, which may have been the reason behind the decreased uptake of the PIC score proforma as clinicians are unlikely to fill out a paper proforma in a predominantly electronic record system.

## Conclusions

The diagnosis of CWI remains challenging especially in settings with limited availability of gold standard diagnostic modality. A structured education programme and the use of the modified PIC score to identify suspected CWI can reduce the time taken to obtain CT and the proportion of missed diagnoses of CWI in ED. Further research is required to validate the use of the modified PIC score for this purpose or to develop a bespoke diagnostic tool for CWI in the ED.

## Figures and Tables

**Figure 6:**
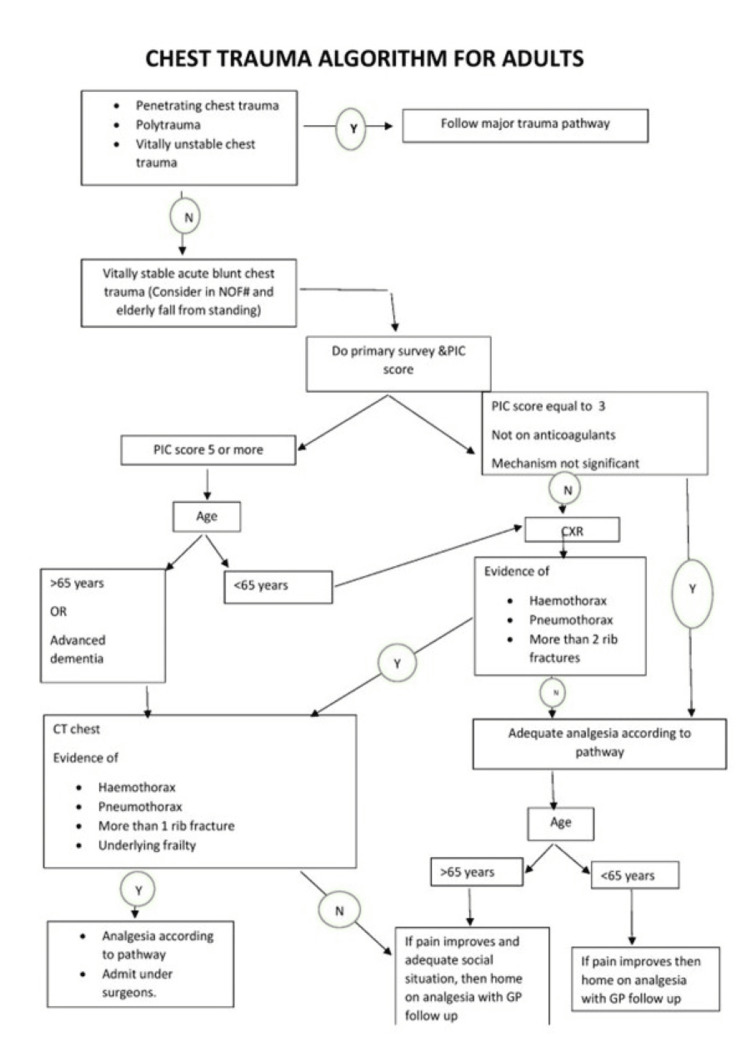
Chest trauma algorithm for adults Image Credit: Salman Naeem

**Figure 7:**
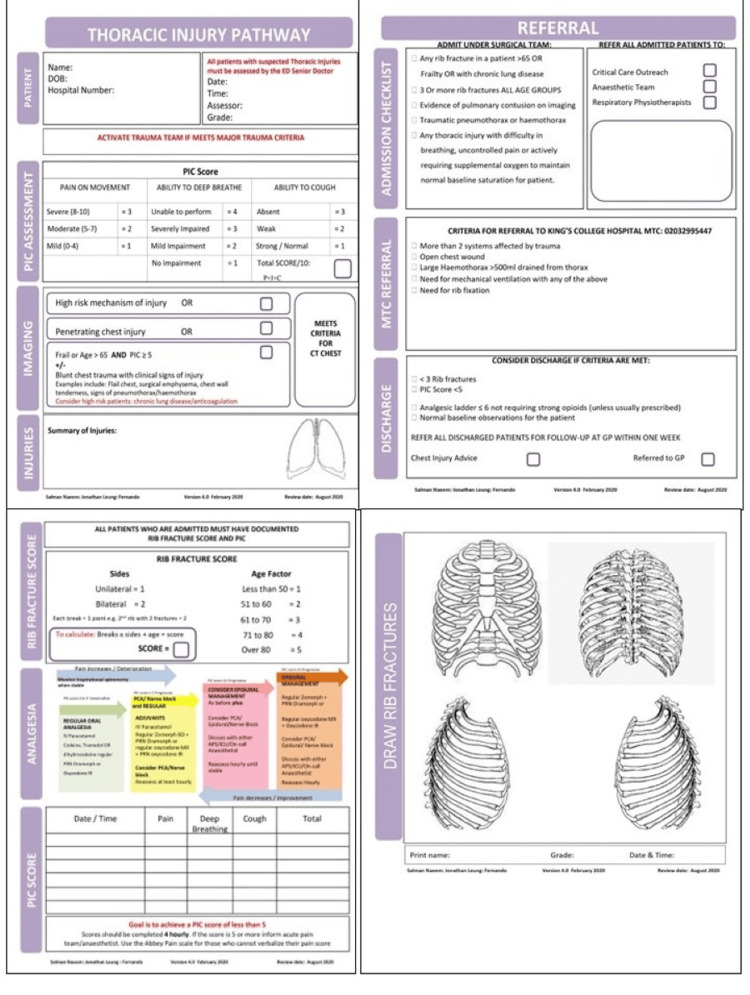
Thoracic injury pathway form Image Credit: Salman Naeem

## Appendices

Appendix A

Appendix B

## References

[1] Pan London Major Trauma System Management of Older TraumaLondon Major Trauma System (2021). London major trauma system. https://www.google.com/url?sa=t&source=web&rct=j&opi=89978449&url=https://www.c4ts.qmul.ac.uk/downloads/pan-london-major-trauma-system-management-of-older-trauma.-third-editionapril-2021.pdf&ved=2ahUKEwjR29qlroyFAxX9VmwGHdr5BxAQFnoECA4QAQ&usg=AOvVaw0B3TPBv7utsrjF6PTuTQA-.

[2] (2022). Falls: applying all our health. Office for Health Improvement.

[3] Banerjee J, Baxter M, Coats T (2017). Major trauma in older people. https://www.google.com/url?sa=t&source=web&rct=j&opi=89978449&url=https://www.gmccmt.org.uk/wp-content/uploads/2019/11/Major-Trauma-in-Older-People-2017-1.pdf&ved=2ahUKEwjIuqzhsYyFAxXZb2wGHScDDRgQFnoECBsQAQ&usg=AOvVaw24zSwi7wK1G13Nr_1SychN.

[4] Battle CE, Hutchings H, Evans PA (2012). Risk factors that predict mortality in patients with blunt chest wall trauma: a systematic review and meta-analysis. Injury.

[5] Bulger EM, Arneson MA, Mock CN, Jurkovich GJ (2000). Rib fractures in the elderly. J Trauma.

[6] Victorino GP, Chong TJ, Pal JD (2003). Trauma in the elderly patient. Arch Surg.

[7] Todd SR, McNally MM, Holcomb JB (2006). A multidisciplinary clinical pathway decreases rib fracture-associated infectious morbidity and mortality in high-risk trauma patients. Am J Surg.

[8] Dillon DG, Rodriguez RM (2021). Screening performance of the chest X-ray in adult blunt trauma evaluation: is it effective and what does it miss?. Am J Emerg Med.

[9] Battle C, Hutchings H, Lovett S (2014). Predicting outcomes after blunt chest wall trauma: development and external validation of a new prognostic model. Crit Care.

[10] Rodriguez RM, Anglin D, Langdorf MI (2013). NEXUS chest: validation of a decision instrument for selective chest imaging in blunt trauma. JAMA Surg.

[11] Terry SM, Shoff KA, Sharrah ML (2021). Improving blunt chest wall injury outcomes: introducing the PIC score. J Trauma Nurs.

[12] Ogrinc G, Davies L, Goodman D, Batalden P, Davidoff F, Stevens D (2016). SQUIRE 2.0 (Standards for QUality Improvement Reporting Excellence): revised publication guidelines from a detailed consensus process. BMJ Qual Saf.

[13] (2018). Advanced trauma life support : student course manual. https://search.worldcat.org/title/Advanced-trauma-life-support-:-student-course-manual/oclc/1042565974.

[14] Aziz S, Bottomley J, Mohandas V, Ahmad A, Morelli G, Thenabadu S (2020). Improving the documentation quality of point-of-care ultrasound scans in the emergency department. BMJ Open Qual.

[15] Maxwell CA, Mion LC, Dietrich MS (2012). Hospitalized injured older adults: clinical utility of a rib fracture scoring system. J Trauma Nurs.

[16] Tripepi G, Jager KJ, Dekker FW, Zoccali C (2010). Selection bias and information bias in clinical research. Nephron Clin Pract.

[17] Prestige E, Stander J, Wei Y (2022). Covid lockdowns in the UK: estimating their effects on transmission. Signif (Oxf).

